# Link Prediction in Heterogeneous Information Networks: Improved Hypergraph Convolution with Adaptive Soft Voting

**DOI:** 10.3390/e28020230

**Published:** 2026-02-16

**Authors:** Sheng Zhang, Yuyuan Huang, Ziqiang Luo, Jiangnan Zhou, Bing Wu, Ka Sun, Hongmei Mao

**Affiliations:** School of Information Engineering, Nanchang Hangkong University, Nanchang 330063, China; 2304081200001@stu.nchu.edu.cn (Y.H.); 2304085410004@stu.nchu.edu.cn (Z.L.); 2404085404312@stu.nchu.edu.cn (J.Z.); 2404085410302@stu.nchu.edu.cn (B.W.); 70308@nchu.edu.cn (K.S.); maohongmei@nchu.edu.cn (H.M.)

**Keywords:** heterogeneous information networks, hypergraph convolutional neural network, soft-voting ensemble strategy, link prediction

## Abstract

Complex real-world systems are often modeled as heterogeneous information networks with diverse node and relation types, bringing new opportunities and challenges to link prediction. Traditional methods based on similarity or meta-paths fail to fully capture high-order structures and semantics, while existing hypergraph-based models homogenize all high-order information without considering their importance differences, diluting core associations with redundant noise and limiting prediction accuracy. Given these issues, we propose the VE-HGCN, a link prediction model for HINs that fuses hypergraph convolution with soft-voting ensemble strategy. The model first constructs multiple heterogeneous hypergraphs from HINs via network frequent subgraph pattern extraction, then leverages hypergraph convolution for node representation learning, and finally employs a soft-voting ensemble strategy to fuse multi-model prediction results. Extensive experiments on four public HIN datasets show that the VE-HGCN outperforms seven mainstream baseline models, thereby validating the effectiveness of the proposed method. This study offers a new perspective for link prediction in HINs and exhibits good generality and practicality, providing a feasible reference for addressing high-order information utilization issues in complex heterogeneous network analysis.

## 1. Introduction

Complex systems in the real world—ranging from individual interactions in social networks and species symbiosis in ecosystems to stock market fluctuations and Internet information flows—exhibit diverse behavioral patterns and time-varying dynamics through multi-level and multi-scale interactions [1]. Their complexity stems from the diversity of node and edge types, as well as the resulting complex structures and rich semantics, which are referred to as HINs in a broad sense and have attracted extensive attention from the academic community [2]. Link prediction on HINs aims to predict missing entities or relationships based on known entities and relationships [3,4].

Currently, link prediction methods on HINs can be mainly categorized into three types: similarity-based methods [5,6], meta-path-based [7] methods, and machine learning-based methods. Lyu L Y et al. [8] classified similarity-based link prediction metrics into local information-based, path-based, and random-walk-based similarity metrics from the perspective of network structure. Local metrics such as Common Neighbors (CN), Jaccard [9], and Adamic–Adar (AA) [10] evaluate similarity through shared neighborhoods. Global metrics such as Katz [11] and local path (LP) aggregate global information through path weight attenuation, performing better in dense networks [12]. To address the defect that link prediction methods on homogeneous networks ignore the semantic information provided by the network, many scholars have proposed meta-path-based similarity methods by integrating meta-paths to improve the accuracy of link prediction on HINs. Sun et al. [13] calculated the similarity of nodes with the same type through symmetric meta-paths, solving the problem of similarity measurement for homogeneous nodes but ignoring attribute information. Shi et al. [14] proposed a bidirectional random-walk-based method to compute the probability of node pairs meeting at intermediate nodes along meta-paths, supporting similarity measurement for arbitrary node types and achieving cross-type node similarity (e.g., author-conference associations) for the first time. However, it suffers from high computational complexity and poor scalability to large-scale networks. Zhao Y H et al. [15] utilized the CBOW model to learn meta-path weights and weighted fuse multiple paths, supporting similarity calculation for arbitrary node types. Li Z Y et al. [16] combined random walk serialization and unsupervised learning to map node structural features into continuous low-dimensional vectors, enabling efficient link prediction for large-scale networks. While meta-path-based methods can intuitively reflect high-order information, they require manual design of effective meta-paths, over-rely on prior knowledge, and only capture path patterns while ignoring global structures, thus resulting in the loss of high-order information. Following the successful application of deep learning and graph neural networks (GNNs) in various graph tasks, supervised learning has become the mainstream research direction. For example, Li et al. [17] constructed a bidirectional deep structure to capture hierarchical network information, introduced l_2,1_-norm to resist noise, and fused local and global features through kernel functions. Liu et al. [18] proposed the DHNR algorithm, which obtains node representations by partitioning temporal subspaces of node neighborhoods, sampling time-weighted meta-path sequences, integrating neighborhood information, and learning spatiotemporal context information. Experimental validation on real datasets showed that it outperforms baseline methods in node classification, clustering, and visualization tasks. Deep learning-based link prediction algorithms for HINs effectively capture complex semantic relationships between multi-type nodes and edges by integrating technologies such as GNNs, attention mechanisms, and representation learning. They have been widely applied in the field of graph analytics and have innovatively optimized and improved the performance of tasks such as node classification [19], prediction [3], and visual analytics [20]. Fan et al. [21] proposed the Path-Aware Multi-Scale Heterogeneous Graph Neural Network (PM-HGNN), which addresses issues such as meta-path redundancy and insufficient utilization of global information.

However, the aforementioned algorithms and models often struggle to capture the ubiquitous collective high-order relationships in reality, such as multi-party collaboration, tripartite transactions, or indirect regulation among species [22]. The significance of high-order interactions has been validated by numerous studies, with applications including drug combination side effect prediction and the understanding of dependency relationships in neuronal avalanche dynamics. In such contexts, there is a need to introduce more expressive relationship models.

A hypergraph [23,24] characterizes high-order relationships via hyperedges that can connect an arbitrary number of nodes, and thus possesses inherent advantages in depicting complex associations. If we can ingeniously construct traditional HINs by incorporating high-order information—treating sets of nodes with specific associations as individual hyperedges—studying HINs from the perspective of hypergraph theory will facilitate more effective exploration of high-order adjacency relationships within the network [25].

In summary, existing research on link prediction methods for heterogeneous networks has analyzed node interactions from multiple perspectives, yet it has not yet fully explored and conducted in-depth analysis on the high-order features of nodes. To address this issue, this study proposes a novel link prediction model for heterogeneous information networks based on a voting ensemble strategy and hypergraph convolution (VE-HGCN). Specifically, the model first extracts multiple distinct FSPs to map the heterogeneous information network into hyperedges, thereby constructing a multi-hypergraph. This approach not only preserves the heterogeneity of the original network but also captures high-order relational information. Subsequently, a hypergraph convolutional neural network (HGCN) is adopted to learn node representations for edge existence prediction. Finally, integrating the voting ensemble strategy from ensemble learning, the model accounts for the differences in high-order information among the constructed heterogeneous hypergraphs and conducts their importance analysis. The main innovations and contributions of this paper are summarized as follows:

(1) We propose a scalable frequent subgraph pattern extraction algorithm. It leverages a random subgraph sampling strategy addressing the limitations of existing hypergraph construction methods that rely on predefined network motifs. Its complexity is significantly lower than that of exhaustive enumeration methods, enabling the algorithm to handle large-scale heterogeneous networks while ensuring the extraction of high-frequency, meaningful FSPs for hypergraph construction.
(2) We proposed an improved hypergraph-based link prediction model via a soft-voting ensemble strategy. This model effectively integrates the HGCN with the ensemble strategy, enabling it to not only learn heterogeneous semantics and high-order structural information from the built multi-view heterogeneous hypergraphs to generate discriminative node embeddings but also adaptively calibrate the ensemble weights of base models to evaluate the importance of such differentiated embeddings, thus significantly improving link prediction accuracy and making it well-suited for highly heterogeneous networks.

The rest of this paper is organized as follows: In Section 2, we present the problem formulation and relevant definitions, including key concepts such as heterogeneous information networks, FSPs, hypergraphs, and ensemble strategies, laying a theoretical basis for the proposed model. Section 3 describes the overall framework and detailed implementation of the VE-HGCN method, elaborating on the three core modules: heterogeneous hypergraph construction, hypergraph convolution-based feature learning, and adaptive soft-voting ensemble strategy. Section 4 is the experimental section, including dataset descriptions, baseline method settings, experimental configurations, and in-depth analysis of experimental results, ablation experiments, and FSP validity studies. Section 5 summarizes the entire paper, highlighting the contributions of the proposed method and outlining future research directions.

## 2. Preliminaries

This section first presents the relevant definitions employed in the proposed model, including heterogeneous information networks, hypergraphs, and voting ensemble strategies, and then provides the problem formulation for link prediction in heterogeneous information networks that integrates soft-voting ensemble strategy with hypergraph convolutional neural networks.

### 2.1. Related Definition

#### 2.1.1. Heterogeneous Information Network (HIN)

**Figure 1:**
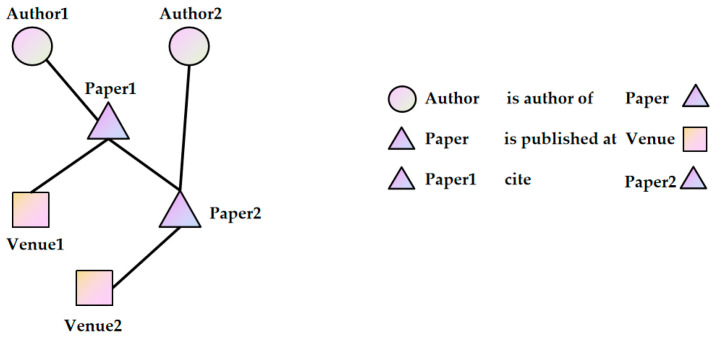
A simple heterogeneous information network.

Let a graph $G=(V,E,{\;T}_{N},T_{E})$, where V represents the vertex set, E represents the edge set, ${\;T}_{N}$ represents the node type set, and $T_{E}$ represents the edge type set. A HIN is a graph containing multiple types of nodes or multiple types of edges. That is, G is a heterogeneous information network if $|T_{E}|>1$ or $|T_{N}|>1$; otherwise, it is a homogeneous information network. The heterogeneous information network illustrated in Figure 1 consists of three types of nodes, namely, author, paper, and venue.

#### 2.1.2. Frequent Subgraph Pattern (FSP)

In complex network analysis, Milo [26] et al. originally defined network motifs as subgraph patterns that are statistically overrepresented compared to randomized null models. Inspired by this, in this paper, we adopt a concept called frequent subgraph patterns, which refer to connected subgraph patterns that occur frequently in the network based on absolute frequency counts. Unlike the statistical definition of motifs, our frequent subgraph patterns do not require comparison with null models and are extracted through random sampling for computational efficiency. This terminology distinction is important: while both concepts capture recurring structural patterns, our approach focuses on high-frequency patterns discovered through sampling rather than statistically significant patterns relative to random graphs.

#### 2.1.3. Hypergraph

**Figure 2:**
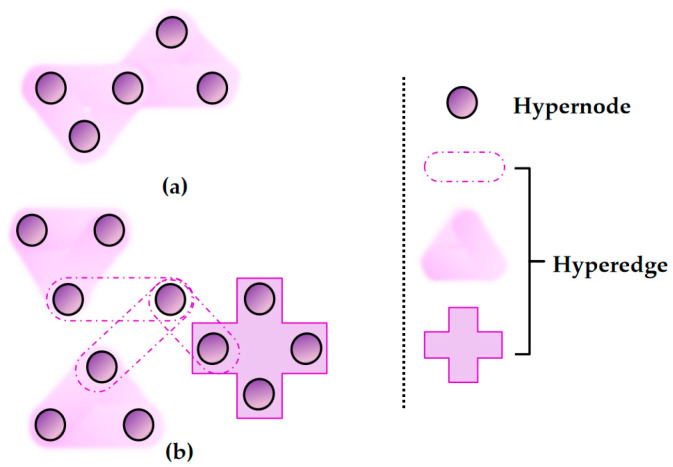
A simple example of two different types of hypergraphs. (**a**) 3-uniform hypergraph; (**b**) non-uniform hypergraph.

A hypergraph H is composed of a vertex set and a hyperedge set, which can be denoted as H=(V,E). Herein, $V=\{v_{1},v_{2},\ldots ,v_{n}\}$, represents the vertex set consisting of n vertices in H, and $E=\{e_{1},e_{2},\ldots ,e_{m}\}$ represents the hyperedge set consisting of m hyperedges. Each arbitrary hyperedge $e_{i}$ is composed of several vertices, and |$e_{i}|$ represents the size of the hyperedge, i.e., when |$e_{i}|=2$ holds for all hyperedges $e_{i}\in E$, the hypergraph is reduced to a conventional graph. A hypergraph is called a uniform hypergraph if all hyperedges in it have the same size. Figure 2 presents a simple illustration of two different types of hypergraphs.

- Incidence Matrix

A single hypergraph can be represented by an incidence matrix $A\in R^{N\times M}$, where *N* represents the number of vertices and *M* represents the number of hyperedges, and the definition of each element in A is given by Equation (1).

(1) $$a_{ij}=\left\{\begin{array}{ll} 1, & if\;v_{i}\in e_{j}\\ 0, & otherwise \end{array}\right.\left(i\in [1\;,N],\;j\in [1,\;M]\right)$$

where $a_{ij}$ indicates that hyperedge $e_{j}$ contains $v_{i}$, and 0 otherwise.

- Hyperedge Degree Matrix

The hyperedge degree matrix $D_{e}\in \times R^{M\times M}$ is a diagonal matrix, and its calculation method is given by Equation (2), which corresponds to the number of vertices m contained, i.e., the size of the hyperedge.

(2) $$D_{e}(m,m)=\sum_{i=1}^{N}H_{im}$$

- Vertex Degree Matrix

The vertex degree matrix $D_{v}\in \times R^{N\times N}$ is a diagonal matrix, and its calculation method is given by Equation (3).

(3) $$D_{v}(n,n)=\sum_{j=1}^{M}H_{nj}w_{j}$$

where $w_{m}$ is the weight of the j-th hyperedge.

#### 2.1.4. Ensemble Strategy

The ensemble strategy, which serves as the combination rule or fusion mechanism in ensemble learning, refers to integrating the prediction results of multiple base models through specific methods to improve the overall performance of the model, reduce variance and bias, or enhance generalization ability. Ensemble strategies are mainly divided into two categories: averaging methods and voting methods.

- Arithmetic Mean

This method directly calculates the arithmetic mean of multiple prediction results, and its calculation method is given by Equation (4).

(4) $$F(x)=\frac{1}{K}\sum_{i=1}^{K}f_{i}(x)$$

where F(x) corresponds to the prediction result of the x-th base learner, and K is the number of base learners involved in the prediction.

- Weighted Mean

This method first assigns weights to different base learners, and its calculation formula is given by Equation (5).

(5) $$F(x)=\sum_{i=1}^{K}{w_{i}f}_{i}(x)$$

where $w_{i}$ is the weight assigned to the x-th base learner, which satisfies the condition $\sum_{i=1}^{K}w_{i}=1$.

- Hard Voting

Based on the principle of majority voting, each base model outputs a class prediction, and the class with the most votes is selected as the final result. For example, if three classifiers predict the classes A, A, and B, the final result is A.

- Soft Voting

Soft Voting is a prevalent ensemble fusion strategy in machine learning, primarily tailored for classification tasks. It performs weighted voting based on the probability distributions predicted by base learners. For example, consider a binary classification problem with two classes, A and B. Model 1 predicts the probability of class A as 0.8 and class B as 0.2; Model 2 predicts the probability of class A as 0.6 and class B as 0.4; and Model 3 predicts the probability of class A as 0.4 and class B as 0.6. The arithmetic mean probability of class A is calculated as (0.8+0.6+0.4)/3=0.6, and that of class B is (0.2+0.4+0.6)/3=0.4. Thus, class A is selected as the classification result.

### 2.2. Problem Description

Given a heterogeneous information network $G=(V,E,T_{N},T_{E})$, $|T_{N}|>1,\;|T_{E}|>1$. For the sake of conciseness, in this work, we assume that the edge type between two nodes is uniquely determined by their node types, which means knowing the node types of a candidate pair is sufficient to determine the corresponding edge type. The task of link prediction is to predict the existence of edges between nodes based on known information, where all nodes V are observed during both training and testing. In this work, link prediction was performed jointly over all edge types. For each edge type ${r\in T}_{E}$ connecting node types ${(t}_{i},t_{j})$, the positive sample set consists of existing edges: $E_{r}^{+}=\{(u,v)\;|\;(u,v)\in E,\;\phi (u,v)=r\}$. To ensure fair evaluation, negative samples were drawn exclusively from type-feasible nodepairs: $E_{r}^{-}=\{(u,v)\;|\;(u,v)\notin E,\;\tau (u)=t_{i},\;\tau (v)=t_{j}\}$. *ϕ* denotes the edge type mapping function, and τ represents the node type mapping function. This constraint excludes type-infeasible pairs, thereby avoiding artificially easy classification. We adopted a 1:1 negative sampling ratio and fixed the negative samples throughout training for reproducibility. Positive and negative samples from every relation type were pooled into a single training/test set. Specifically, the overall positive set is $E^{+}={⋃}_{r\in T_{E}}E_{r}^{+}$, and the overall negative set is $E^{-}={⋃}_{r\in T_{E}}E_{r}^{-}$. Given the training graph $G_{train}=(V,E_{train})$, where $E_{train}\subset E,$ we first extracted the set of FSP instances $M^{\left(K\right)}$ on $G_{train}$ using a FSP extraction algorithm then constructed the corresponding set of hypergraphs $H_{M_{k}}^{\left(K\right)}\;(k\;\in \;[1,\;K\;])$ from different network FSPs. A VE-HGCN model was designed to firstly learn high-order node features from each constructed hypergraph and then get a set of prediction probability results $P^{\left(K\right)}$. Finally the soft-voting ensemble strategy was employed to obtain the final link prediction results $P_{final}$.

## 3. Model

### 3.1. Overall Architecture

**Figure 3:**
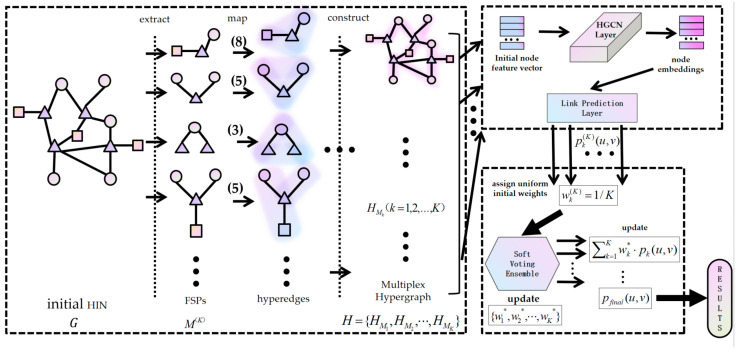
The VE-HGCN model framework. The numbers in parentheses on the arrows denote the occurrence frequency of each FSP, and the asterisk (*) indicates the optimized weights learned on the validation set.

This section introduces the proposed VE-HGCN model based on the soft-voting ensemble strategy and HGCN. As illustrated in Figure 3, the overall architecture of the VE-HGCN model integrates three core modules: hypergraph construction, hypergraph convolution, and soft-voting ensemble strategy.

Specifically, the heterogeneous hypergraph construction module processes the raw input heterogeneous information network. It first extracts the top K distinct network FSPs ranked by occurrence frequency using an extraction algorithm, then maps these distinct FSPs to hyperedges and constructs the corresponding K hypergraphs. This design not only preserves the high-order properties of the network but also retains its heterogeneity. For the feature learning, it starts by initializing node feature vectors for the constructed multiplex hypergraphs, considering that each hyperedge is mapped from a specific network FSP, which inherently contains multiple interconnected nodes. This concatenation strategy effectively encodes the node-level information within the hyperedge, laying a foundation for capturing high-order relational features. The hyperedge features are implicitly handled by the incidence matrix and degree matrices emerging through the message passing process. Subsequently, a hypergraph convolutional neural network is applied to each hypergraph independently for feature learning: the network aggregates high-order topological and attribute information from the hypergraph structure to refine node representations, which are then fed into a prediction layer to perform preliminary link prediction tasks. Eventually, this module outputs K sets of prediction probabilities, where each set corresponds to the link existence probability between target nodes derived from one of the K hypergraphs. The voting ensemble module leverages a soft-voting ensemble strategy to integrate the K sets of preliminary link prediction probabilities obtained from the feature learning module. Given that different hypergraphs capture complementary high-order structural patterns of the original heterogeneous network, simply relying on a single hypergraph’s prediction may lead to biased results due to incomplete feature representation. The soft-voting strategy addresses this limitation by computing the weighted average of the K probability distributions with uniformly assigned initial weights to ensure fairness in the early stage of integration. It then selects the class with the highest aggregated probability as the final prediction result. This improvement effectively fuses the complementary information from multiple hypergraphs, reduces the prediction bias caused by single-model limitations, and thereby enhances the accuracy and generalization ability of link prediction.

### 3.2. Construction of Heterogeneous Hypergraphs

#### 3.2.1. Frequent Subgraph Pattern Extraction

Existing methods for constructing hypergraphs from heterogeneous information networks either enumerate all node subgraphs for isomorphism judgment or rely on predefined network FSPs. These approaches are prohibitively challenging for complex, large-scale datasets of diverse heterogeneous information networks. Enumerating all nodes to construct FSPs incurs exponential time complexity, leading to incalculable computational overhead. In addition, the diversity of node and relation types generates an excessive number of potential FSPs, and the variability of FSPs in heterogeneous graphs renders the description of universal FSPs impractical. To address this issue, we propose a universal network FSP extraction algorithm applicable to any dataset, based on the fundamental definition of an FSP, which is grounded in random subgraph sampling. The algorithm proceeds as follows: first, seed nodes are selected on the heterogeneous graph with weights assigned according to node degree, and a hybrid strategy of neighborhood priority and global jump is adopted to gradually expand subgraphs, yielding candidate subgraphs of a specified size. Subsequently, a connectivity check is performed with only connected subgraphs retained. For each subgraph, isomorphism-invariant FSPs are constructed based on its structural adjacency and node types, and their occurrence frequencies are accumulated in a hash table while representative instances are preserved. Finally, the top-K FSPs of each size are selected by frequency and fed into subsequent hypergraph construction as explicit patterns of high-order relations. Algorithm 1 summarizes the complete process of network FSP extraction. The Subfunction ${sample}_{subgraph}$ and ${code}_{FSP}$ used in Algorithm 1 is illustrated in Algorithm 2 and after.
**Algorithm 1:** Heterogeneous Network Frequent Subgraph Pattern Extraction**Inuput:** G: HIN; ${size}_{FSP}$: Target size of FSPs; K: Number of top *K* FSPs to extract.**Output:** ${instances}_{FSP}$: Set of top K FSP instances with the highest occurrence frequency.**Function:** ${sample}_{subgraph}$ (); ${code}_{FSP}$ ().1: **Initialize** ${sampled}_{nodes}$, *Frequency*, ${node}_{degree}$, ${instances}_{FSP}$, ${node}_{neighbors}$ ← empty set; ${num}_{samples}$=100,000;2: // **Step 1:** Preprocessing—Calculate node degrees and neighbor sets (O(|*V| + |E*|))3: **For** each node *v* in *V* do4:  ${node}_{degree}$*[v] ← |{e ∈ E | v ∈ e}|* // Compute total degree of node v5:  ${node}_{neighbors}$*[v] ← {u ∈ V | (v,u) ∈ E or (u,v) ∈ E*} // Neighbor set (including edge types)6: **End for**7: // **Step 2:** Random subgraph sampling and FSP statistics (O(${num}_{samples}$
*×*
${motif}_{size}^{2}$))8: **For** *i* = 1 to ${num}_{samples}$ do // Sample subgraph of target size (core subfunction: ${sample}_{subgraph}$)9:  ${sampled}_{subgraph}$ ← ${sample}_{subgraph}$*(G,*
${size}_{FSP}$*,*
${node}_{degree}$*,*
${node}_{neighbors}$*)*10:  **If not is_connected**(${sampled}_{subgraph}$) then // Connectivity check: only process connected subgraphs11:   Continue // Skip disconnected subgraphs to avoid invalid FSPs12:  **End if**13: // Update frequency and instance mapping14: f ← ${code}_{FSP}$(${sampled}_{subgraph}$)15:  **If** *f* not in ${Frequency}_{\mathrm{FSP}}$ then16:   ${Frequency}_{\mathrm{FSP}}$*[f] ← 0*17:  **End if**18:  ${Frequency}_{\mathrm{FSP}}$*[f]* += 119:  **If** *f* not in ${instances}_{FSP}$ then20:   ${instances}_{FSP}$*[f] ←* ${sampled}_{subgraph}$ // Store representative instance21:  **End if**22: **End for**23: // **Step 3**: Filter high-frequency FSPs (**O***(M log M*), *M* = number of unique *FSPs ≪* ${num}_{\mathrm{samples}}$)24: ${sorted}_{\mathrm{FSPs}}$ ← Sort Frequency in descending order of values25: ${instances}_{FSP}$ ← Take first *K* entries from ${sorted}_{\mathrm{FSPs}}$
26: // **Step 4**: Output results27: **Return** ${instances}_{FSP}$

**Algorithm 2:** Function: Core Subfunction: ${sample}_{subgraph}$ (*G*, ${size}_{FSP}$, ${node}_{degree}$, ${node}_{neighbors}$)1: // **Step 1:** Weighted sampling of seed node (higher degree → higher sampling probability)2: ${total}_{degree}$ ← Sum(${node}_{degree}$*[v]* for *v* in *V*)3: ${seed}_{node}$ ← Randomly select *v* ∈ *V* with probability ${node}_{degree}[v]\;/\;{total}_{degree}$
4:${\;sampled}_{nodes}$ ← {${seed}_{node}$} // Initialize sampled node set5: // **Step 2:** Neighborhood-priority expansion + global jump, avoid local over-sampling6: **While** |${sampled}_{nodes}$*| <*
${size}_{FSP}$ do // $p_{expand}$: Expansion probability from neighbors of sampled nodes7:  **If** Random(0,1) < $p_{expand}$ and $(\cup_{v\in sampled\_nodes}node\_neighbors[v]\backslash {sampled}_{nodes})\neq \emptyset$ then8:    ${candidate}_{nodes}$*←*$\cup_{v\in {sampled}_{nodes}}{node}_{neighbors}[v]\backslash {sampled}_{nodes}$
9:    ${next}_{node}$ ← Randomly select *u* ∈ ${candidate}_{nodes}$
10:  Else:11:   ${next}_{node}$ ← Randomly select *u* ∈ *V* \ ${sampled}_{nodes}$ // global random jump: ensure sampling diversity12:  **End if**13:  ${sampled}_{nodes}$.add(${next}_{node}$)14: **End while**15: // **Step 3**: Extract complete subgraph info (nodes + edges + types)16: ${sampled}_{edges}$ ← {*e* ∈ *E* | *e* connects two nodes in ${sampled}_{nodes}$}17: ${sampled}_{subgraph}$ ← (${sampled}_{nodes}$*,*
${sampled}_{edges}$*,*
$\tau ({sampled}_{nodes})$*,*
$\varphi ({sampled}_{edges})$)18: **Return** ${sampled}_{subgraph}$


The function ${code}_{FSP}$(${sampled}_{subgraph}$) generates a type-aware heuristic signature for each sampled subgraph, aiming to produce identical codes for structurally equivalent subgraphs with the same node and edge type configurations. The process is as follows: First, sort the nodes in the sampled subgraph by their type labels τ(v). To resolve ordering ambiguity among nodes sharing the same type, a secondary sorting criterion is applied: within each type group, nodes are sorted by their subgraph-internal degree, i.e., the number of edges incident to the node within the sampled subgraph in descending order; if degrees are also tied, nodes are further sorted by the lexicographically sorted list of their neighbor types within the subgraph. This yields a refined node ordering: $v_{\pi (1)},v_{\pi (2)},\ldots ,v_{\pi (k)}$, where π is the sorting permutation. Second, for the sorted nodes, an adjacency representation is constructed that includes edge type information: ${adj}_{matrix}[i][j]=\phi (v_{\pi (i)},v_{\pi (j)})$ if an edge exists between $v_{\pi (i)}$ and $v_{\pi (j)}$ and ${adj}_{matrix}[i][j]=0$ otherwise. Third, the sorted node types are concatenated to form the type sequence: $type_{seq}=[\tau (v_{\pi (1)}),\tau (v_{\pi (2)}),\ldots ,\tau (v_{\pi (k)})].$ Finally, the hash-based pattern code is computed as $m_{code}=hash(type_{seq}∥flatten(adj_{matrix}))$, where ‖ denotes concatenation and flatten(⋅) converts the adjacency matrix to a 1D array.

For example, consider a 3-node subgraph from the DBLP dataset with nodes $\left\{{Author}_{1},{Paper}_{1},{Author}_{2}\right\}$ and edges $\left\{({Author}_{1},writes,{Paper}_{1}),({Author}_{2},writes,{Paper}_{1})\right\}$. After sorting by node type (author < paper), the canonical representation is as follows: Sorted nodes: $\left[{Author}_{1},{Author}_{2},{Paper}_{1}\right]$; Type sequence: [A,A,P]; Adjacency matrix with edge types: $\left[\begin{array}{ccc} 0 & 0 & writes\\ 0 & 0 & writes\\ writes & writes & 0 \end{array}\right];\;m_{code}=hash([A,A,P,0,0,w,0,0,w,w,w,0])$.

This design not only adapts flexibly to diverse HIN datasets without relying on predefined pattern templates but also achieves efficient computation with a time complexity of $O\left({num}_{samples}\times {size}_{FSP}^{2}+M\log M\right)$, where ${num}_{samples}$ is the number of sampled subgraphs, ${size}_{FSP}$ is the target size of subgraph patterns, and M is the number of unique patterns discovered.

We note that this heuristic procedure does not guarantee strict isomorphism invariance in all cases. When multiple nodes of the same type have identical subgraph-internal degrees and identical neighbor type distributions, the ordering among them remains ambiguous, which may lead to distinct codes for isomorphic subgraphs (false negatives) or, conversely, identical codes for non-isomorphic subgraphs (hash collisions). However, such pathological cases are rare in practice for the small subgraph sizes (${size}_{FSP}$ ≤ 5) considered in this work because the combination of type labels, degree ordering, and neighbor type sorting resolves most ambiguities. Moreover, since the downstream task only requires identifying the top-*K* most frequent patterns, occasional coding errors have negligible impact on the final pattern ranking and hypergraph construction quality. We empirically observed consistent experimental results across multiple runs, confirming the practical robustness of this heuristic encoding.

#### 3.2.2. Hypergraph Construction

As presented in Section 3.2.1, the set of K FSPs in the HIN has been identified via the proposed extraction algorithm. To construct hypergraphs, we first map each FSP $M_{t}(t=(1,K))$ to a hyperedge, thereby building a corresponding single hypergraph $H_{M_{t}}(V,E_{M_{t}})$ for each FSP $M_{t}(t=(1,K))$, specifically, $H_{M_{t}}(V,E_{M_{t}})$, where V denotes the entire node set of the original HIN and $E_{M_{t}}$ is the hyperedge set formed by mapping all sampled instances of $M_{t}(t=(1,K))$ to hyperedges. However, constructing a hypergraph based on a single FSP is insufficient for semantically rich heterogeneous networks as an individual FSP can only capture partial high-order structural semantics. Therefore, we further match the hypergraph instances of all K FSPs in the HIN and integrate these K single hypergraphs into a unified multiplex hypergraph ${H=\{H}_{M_{1},\;}\;H_{M_{2},}\;{\;H}_{M_{3}},\ldots ,\;H_{M_{K}}\}$ by different FSPs, laying a foundation for subsequent feature learning.

### 3.3. VE-HGCN Model

#### 3.3.1. Hypergraph Convolutional Neural Network (HGCN)

In recent years, with the continuous deepening of research on neural networks, their application scope has been continuously expanded, and the field of hypergraph learning has also witnessed the integration of neural network methods. In hypergraph learning, spectral analysis-based methods implicitly extend GCNs to hypergraphs by virtue of the Fourier transform and wavelet transform, supported by a solid mathematical foundation, thus achieving strong interpretability. Essentially, HGCNs are obtained by explicitly or implicitly extending GCNs to hypergraphs or by introducing attention mechanisms to design convolution operations suitable for hypergraphs.

Bruna et al. [27] were the first to design spectral convolution operators on graphs using the convolution theorem and proposed Spectral CNN, but it suffers from high computational complexity. To address this issue, Defferrard [28] approximated the convolution kernel with K-th-order Chebyshev polynomials, where the graph convolution operator depends on the K-th-order neighbors of vertices. To simplify parameters, Kipf et al. [29] proposed a GCN that only uses 1st-order Chebyshev polynomials to approximate the convolution kernel, which improved the performance of semi-supervised learning on graphs. The convolution layer of the GCN is defined as

(6) $$\begin{array}{c} H^{\left(l+1\right)}=\sigma \left({\tilde{D}}^{-\frac{1}{2}}\tilde{A}{\tilde{D}}^{-\frac{1}{2}}H^{\left(l\right)}W^{\left(l\right)}\right) \end{array}$$

where $\tilde{A}=A+I,$ A denotes the adjacency matrix of the graph, I is the identity matrix, $\tilde{D}$ represents the degree matrix of $\tilde{A}$. The inverse and square root operations in $\tilde{D}$ are used to normalize the structural information in the graph, enabling nodes with different degrees to participate in computations more reasonably. $H^{\left(l\right)}$ denotes the node feature matrix of the l-th layer, which records the feature information of nodes in the graph. σ(⋅) is an activation function (commonly used ones include ReLU, Sigmoid, etc.), which introduces non-linearity into the computation and outputs the input after non-linear transformation to enhance the model’s expressive ability. $W^{\left(l\right)}$ is a learnable parameter matrix, whose values are continuously adjusted through optimization algorithms during model training to learn effective feature patterns in hypergraph data and fit complex relationships and features on the graph. The superscript l represents the parameters corresponding to the l-th layer.

Overall, Equation (6) describes, in a certain layer of the graph neural network, the process of updating the feature representation by performing a series of transformations on the input node feature matrix combined with learnable parameters based on the graph’s structural information, reflected by $\tilde{D}$, $\tilde{A}$, followed by the activation function. This is one of the key steps for graph neural networks to implement node feature propagation, aggregation, and update, enabling the completion of learning tasks on graphs, e.g., node classification and link prediction.

Traditional graph convolutional networks study the properties of graphs through the eigenvalues and eigenvectors of the graph Laplacian matrix. Zhou et al. [30] defined the hypergraph Laplacian matrix, as shown in Equation (7):

(7) $$L_{G}=I-{D_{G}}_{v}^{-\frac{1}{2}}H_{G}W_{G}{D_{G}}_{e}^{-1}H_{G}^{⊤}{D_{G}}_{v}^{-\frac{1}{2}}$$

where $D_{G_{v}}$ is the node degree matrix of the hypergraph G, and $D_{G_{e}}$ is the hyperedge degree matrix. Similarly, the inverse transformation and square root operations are applied to normalize structural information, ensuring that nodes and hyperedges with different degrees participate in computations more reasonably and avoiding computational imbalance caused by degree differences. $W_{G}$ is the weight matrix of the hypergraph, and $H_{G}$ is the incidence matrix of the hypergraph. $L_{G}$ is a positive semi-definite symmetric matrix, and its eigen-decomposition is $L_{G}=U\Lambda U^{⊤}$, where U is the orthogonal eigenvector matrix, $UU^{⊤}=I$, and Λ is the diagonal matrix of eigenvalues.

For node features $x\in R^{N}$, the hypergraph Fourier transform is defined as follows:

1. Forward transform (node domain → frequency domain):

(8) $$\hat{x}(\lambda_{k})=F[x](\lambda_{k})=\sum_{i-1}^{N}{x(i)u}_{k}^{*}(i)$$

where $u_{k}$ is the k-th eigenvector of the hypergraph Laplacian satisfying $L_{G}u_{k}=\lambda_{k}u_{k}$, and $u_{k}^{*}(i)$ is the conjugate of $u_{k}(i$). Essentially, eigenvectors are used as the “Fourier basis” to project node-domain signals into the frequency domain.

2. Inverse transform (frequency domain → node domain):

(9) $$x(i)=F^{-1}\{\hat{x}\}(i)=\sum_{k=1}^{N}{\hat{x}(\lambda }_{k})\mu_{k}(i)$$

The goal of hypergraph convolution is to propagate and aggregate node features along the hyperedge structure. In the spectral domain, convolution can be simplified to point-wise multiplication, which is similar to graph convolution. Let the convolution kernel be *g*; the convolution of the feature x is defined as shown in Equation (10). The convolution kernel corresponds to $\hat{g}=F\{g\}=U^{T}g$ in the spectral domain. Since the eigenvectors of the hypergraph Laplacian are orthogonal, if *g* is assumed to be a smoothing kernel based on the hypergraph structure, and only related to eigenvalues, $\hat{g}$ can be expressed as a diagonal matrix $\hat{G}=diag\left(\hat{g}(u_{1}),\ldots ,\hat{g}(u_{N})\right)$. Combined with the normalized form of the hypergraph Laplacian, the “neighborhood aggregation” of the hypergraph structure can be realized through the complement of $L_{G}$ (i.e., $I-L_{G}={D_{G}}_{v}^{-\frac{1}{2}}H_{G}W_{G}{D_{G}}_{e}^{-1}H_{G}^{⊤}{D_{G}}_{v}^{-\frac{1}{2}}$), denoted as the structural operator *A*. Then the Fourier transform of *A* is $\hat{A}=U^{T}AU=I-\Lambda$.

(10) $$x*g=F^{-1}\left(F(x)⊙F(g)\right)$$

Let the node feature of the current layer be $X^{\left(l\right)}{=P}_{G}^{\left(l\right)}$ (a matrix of size N×C, where C is the feature dimension), and the learnable weight be $\Theta^{\left(l\right)}=\Theta_{G}^{\left(l\right)}$ (a matrix of size $C\times C^{\prime }$), where $C^{\prime }$ is the dimension of the next layer). The feature is first linearly transformed, then the structural operator $X^{\left(l\right)}\Theta^{\left(l\right)}\in R^{N\times C^{\prime }}$, *A·*$X^{\left(l\right)}\Theta^{\left(l\right)}$ is used to aggregate the transformed features to simulate information propagation associated with hyperedges. To enhance non-linear expression, an activation function is finally introduced, and the final hypergraph convolution layer is shown in Equation (11):

(11) $$X_{G}^{\left(l+1\right)}=\sigma \left({D_{G}}_{v}^{-\frac{1}{2}}H_{G}W_{G}{D_{G}}_{e}^{-1}H^{⊤}{D_{G}}_{v}^{-\frac{1}{2}}X_{G}^{\left(l\right)}\Theta_{G}^{\left(l\right)}\right)$$

#### 3.3.2. Feature Construction

Hypergraph convolution can handle hyperedge structures and capture high-order relationships between nodes. Using hypergraph convolutional neural networks to learn the embedding representation of each node, the construction of initial feature vectors is crucial. These features need to enable the neural network to accurately identify the target node pairs and the structural importance of other nodes in the network, which determines what kind of information the model can learn. Constructing initial node feature vectors for heterogeneous hypergraphs needs to consider the following points:

- Handling the diversity of node types;
- Features of hyperedges;
- Structural features;
- Normalization and alignment of different feature vectors.

Considering the above issues, let the initial feature vector of node v in the *k*-th (*k* = 1, 2, …, *K*) hypergraph be $X_{v}^{\left(k\right)}\in R^{2+|T_{N}|}$, which contains topological and semantic information, defined as shown in Equation (12):

(12) $$\begin{array}{c} X_{v}^{\left(k\right)}=\left[x_{v}^{v\_deg},{\;x}_{v}^{e\_deg},\;{one}_{hot}(\tau (v))\right] \end{array}$$

where $x_{v}^{v\_deg}$ denotes the hyper-degree of node *v*, and ${\;x}_{v}^{e\_deg}$ denotes the hyperedge degree of the hyperedges where node *v* is located. If node v is associated with multiple hyperedges, the average of the hyperedge degrees of all associated hyperedges is calculated, and ${one}_{hot}(\tau (v))\in R^{\left|T_{N}\right|}$ denotes the one-hot encoding of node v’s type, where $\left|T_{N}\right|$ is the number of node types. This one-hot representation avoids the arbitrary ordering problem that arises from using scalar type IDs and properly treats node types as categorical variables.

To eliminate the influence of feature dimension differences and ensure the stability of hypergraph convolution training, z-score feature normalization is performed on the initial node feature vector before input to the HGCN. Subsequently, the initial node feature vectors are input into the hypergraph convolution layer for feature learning where node features are propagated to hyperedges and hyperedge information is propagated back to nodes. This is implicitly handled by the incidence matrix and degree matrices, and the link prediction layer predicts the existence probability of edges by concatenating the embedding features of nodes at both ends of the edge.

#### 3.3.3. Voting Ensemble Strategy

While the HGCN enables cross-multi-node feature fusion via hyperedges, allowing node embeddings to incorporate not only information from their direct neighbors but also global attributes of the high-order groups they participate in, different representations of heterogeneous hypergraphs inevitably impact link prediction results. This is because distinct network FSPs have varying high-order information, which exerts differential influences on generating the final heterogeneous network representation. Simply integrating features from different hypergraphs without differentiation will undoubtedly compromise model training outcomes. Diversity is the foundation of performance improvement in ensemble learning, whose key lies in ensuring significant differences among base learners through the adoption of different algorithms, training data subsets, and parameter settings. This section discusses how to leverage a voting ensemble strategy to combine “good and diverse” base models, achieving a synergistic effect 1 + 1 > 2 and enhancing the robustness and accuracy of the overall model.

In Section 3.2, the original heterogeneous network can be constructed into a set of K heterogeneous hypergraphs based on distinct FSPs, denoted as ${H=\{H}_{M_{1},\;}\;H_{M_{2},}\;{\;H}_{M_{3}},\;\ldots ,\;H_{M_{K}}\}$ ($H_{M_{k}}=(V,\;E_{M_{k}})$, k ∈ [1,*K*]). An initial feature vector $x_{i}\in R^{D}$ is assigned to each node to form the initial feature matrix $X\in R^{N\times D}$, where N is the number of nodes, and D is the dimension of initial features. For any node pair (u,v), its input feature is constructed via feature concatenation. For each heterogeneous hypergraph $H_{M_{k}}$, an independent HGCN is built: the initial node features are fed into the hypergraph convolution model in Section 3.3.1, and the semantic features of nodes $Z_{k}\in R^{N\times D^{\prime }}$, where $D^{\prime }$ is the dimension of learned features, are learned through the feature propagation mechanism in Equation (11). For a node pair (u,v), its semantic features $Z_{u,k}\in R^{D^{\prime }}$ and $Z_{v,k}\in R^{D^{\prime }}$ are extracted, and a 2-layer MLP prediction head outputs the probability score of a link existing between this node pair. The calculation is shown in Equation (13):

(13) $$p_{k}(u,v)=\sigma (W_{pred}\cdot (Z_{u,k}⊕Z_{v,k})+b_{pred})$$

where σ(·) denotes the Sigmoid activation function with output range [0,1], $W_{pred}$, $b_{pred}$ are the parameters of the prediction head, and ⊕ represents feature concatenation.

Before training, the dataset is split into a training set $D_{train}$, a validation set $D_{val}$ for weight learning, and a test set $D_{test}$. Based on the training set, the parameters of the HGCN and prediction head for the k-th branch are optimized using the cross-entropy loss function in Equation (14), ensuring each single branch can learn independently:

(14) $$L_{k}=-\sum_{(u,v)\in D_{train}}\left[y(u,v)\mathrm{lo}gp_{k}(u,v)+(1-y(u,v))\mathrm{lo}g\left(1-p_{k}(u,v)\right)\right]$$

Preliminary experiments show that simply averaging the probabilities obtained by training the *K* branches (via Equation (4)) yields only marginal improvements in overall model performance, yet link prediction results still vary. This fully demonstrates that the multiple hypergraphs mapped from different hyperedges differ in importance. Therefore, after the feature learning module using the HGCN, this paper introduces a voting ensemble strategy with adaptive weight updating to enhance the model’s ability to weigh features of different high-order relationships.

Adaptive weights $\left\{{\omega_{1}{,\;\omega }_{2},\;\ldots ,\omega }_{K}\;\right\}$ are assigned to the K branches, satisfying $\omega_{k}\geq 0$ and $\sum_{k=1}^{K}\omega_{k}=1$, where the weight magnitude reflects the semantic importance of the corresponding FSP. The weights are initialized uniformly, and optimal weights are learned by minimizing the ensemble prediction loss on the validation set. The ensemble prediction probability is defined as $p_{ens}(u,v)=\sum_{k=1}^{K}\omega_{k}p_{k}(u,v))$. The optimization objective is to minimize the ensemble cross-entropy loss, as shown in Equation (15). The trained branch weights $\left\{\omega_{1}^{*},\;\omega_{2}^{*},\;\ldots ,\omega_{K}^{*}\right\}$ are then applied to the test set. For any node (u,v) in the test set, the prediction probabilities $\left\{p_{k}(u,v){\}}_{k=1}^{K}\right)$ from all branches are obtained and weighted summed using the optimal weights to derive the final ensemble prediction probability $p_{final}(u,v)=\sum_{k=1}^{K}\omega_{k}^{*}p_{k}(u,v))$.

(15) $${\min\{w_{k}\}L}_{ens}=-\sum_{(u,v)\in D_{val}}\left[y(u,v)\mathrm{lo}gp_{ens}(u,v)+(1-y(u,v))\mathrm{lo}g\left(1-p_{ens}(u,v)\right)\right]$$

The final result is output based on the ensemble probability. A threshold Γ is determined by the optimal F1-Score on the validation set. Specifically, we perform grid search over candidate thresholds *Γ* ∈ {0.1, 0.2, 0.3, 0.4, 0.5, 0.6, 0.7, 0.8, 0.9} and select the threshold that maximizes F1-Score: Γ* = argmax_*Γ* F1(*Dval*, *Γ*), where F1(*Dval*, *Γ*) is the F1-Score computed on the validation set using threshold Γ. For binary classification, if $p_{final}(u,v)$ > Γ*, the prediction result is “link exists”; otherwise, it is “link does not exist”. For link prediction tasks targeting potential links in a ranking setting instead of binary classification, node pairs are sorted in descending order of $p_{final}$, and the Top-*K* node pairs are selected as the prediction results.

## 4. Experiments and Data

This section compares the link prediction performance of the proposed VE-HGCN algorithm with that of baseline models on three types of commonly used real-world datasets and also describes the relevant ablation experiments conducted in this study. To verify the performance of the VE-HGCN in link prediction, four publicly available heterogeneous network datasets with distinct characteristics were selected for experiments, with AUC and F1-Score adopted as the benchmark evaluation metrics. The experiments were conducted on a Linux platform equipped with an A100 80G GPU, with the running environment configured as Python 3.10.13 + PyTorch 2.0.0 + PyTorch-Geometric 2.5.1.

### 4.1. DataSets

The details of each dataset are presented in Table 1. DBLP1 and DBLP2 are small-scale subnetworks extracted from the DBLP dataset; the full DBLP dataset contains over 5 million records, where each record includes metadata, e.g., title, author, venue, and publication year and citation information including the number of citations received by a paper and the papers that cite it, etc. The node types of DBLP1 include papers and authors, while DBLP2 additionally incorporates conference nodes. The IMDB dataset consists of information about movies, with node types corresponding to movie names, release years, genres, and directors. LAST.FM establishes a detailed profile of each user’s musical preferences by recording comprehensive information about the tracks listened to by users, and its node types are users, artists, songs, and albums.

### 4.2. Baseline Methods

Existing methods for link prediction tasks are mainly categorized into unsupervised learning-based and supervised learning-based approaches. For methods that are only applicable to homogeneous networks, the original heterogeneous information network is directly used as input. The following seven baseline algorithms were selected for comparison with the VE-HGCN model in this study:

1. LINE (Large-Scale Information Network Embedding) [31]

This algorithm generates low-dimensional vector representations by preserving the first-order proximity (direct connections) and second-order proximity (shared neighbor structures) of nodes. This method does not take network heterogeneity into account.

2. Metapath2vec [32]

This method is specifically designed for heterogeneous graphs; it guides random walks via meta-paths to learn node embeddings.

3. Meta-GNN (Meta Graph Neural Network) [33]

The Meta-GNN integrates meta-learning with graph neural networks (GNNs), enabling rapid adaptation to new graph structures with a small number of samples.

4. GCN [29]

This method aggregates neighbor information through convolution operations and hierarchically extracts node features.

5. HAN (Heterogeneous Graph Attention Network) [34]

The HAN introduces an attention mechanism into heterogeneous graph embedding to distinguish the weights of different node types and edge types.

6. HGCL (Heterogeneous Graph Contrastive Learning) [35]

This approach applies contrastive learning to heterogeneous graphs for recommendation tasks, preserving both structural proximity and semantic heterogeneity of nodes through contrastive loss.

7. ie-HGCN [36]

The ie-HGCN enhances the traditional HGCN with an interaction-aware mechanism, effectively capturing the implicit interaction correlations between nodes and edges in heterogeneous graphs.

### 4.3. Experimental Setup and Evaluation Metrics

We performed 5 independent runs, each with a different random seed. In each run, the full edge set E comprising all edge types jointly was randomly split into 80% training edges and 20% test edges via stratified sampling that preserved the edge type distribution across splits. A training graph $G_{train}=(V,E_{train})$ was then constructed with only training edges, where all test edges were completely removed from the graph structure to eliminate data leakage before any subsequent operations. Specifically, the frequent subgraph pattern extraction algorithm (Section 3.2.1) was exclusively applied to $G_{train}$, and all extracted patterns along with their frequencies were computed solely based on the topology of $G_{train}$; correspondingly, all hypergraphs were constructed from $G_{train}$, with hyperedges derived only from pattern instances identified within the training graph. Within each run, the training set $E_{train}$ was further partitioned via 5-fold cross-validation: in each fold, 4/5 of the training edges were used for model optimization, and the remaining 1/5 served as a validation set for early stopping and hyperparameter tuning. Link prediction was trained and evaluated jointly over all edge types: positive and negative samples from every relation type were pooled into a single training/test set. The VE-HGCN model was implemented on the PyTorch framework, employing a 2-layer hypergraph convolutional neural network for node feature extraction with convolutional channel sizes configured as 64 and 32. The initial node feature vector (dimension = $2+|T_{N}|$) integrates three components: hyper-degree, average hyperedge degree, and one-hot encoded node type. The extracted node features were further fed into a link prediction module consisting of two fully connected layers with channel sizes of 64 and 2 to output the probability of edge existence between node pairs. The final reported results are the mean ± standard deviation of the 5 independent test set performances (one per run). Table 2 presents the complete hyperparameter configuration of the VE-HGCN model, covering the details of key parameters related to model architecture, training optimization, and data processing.

In link prediction tasks, AUC and F1-Score are important evaluation metrics. AUC focuses on the ranking capability of positive and negative samples without requiring the setting of a classification threshold. It exhibits strong robustness to the highly imbalanced sample distribution in link prediction and can precisely match the characteristics of the similarity scores output by the model, serving as a fundamental and core metric for measuring the overall discrimination ability of the model. In contrast, F1-Score focuses on classification accuracy under a specific threshold, balancing precision and recall through the harmonic mean. It can verify the actual classification performance of the model and make up for the limitation that AUC does not reflect specific classification results, which is particularly suitable for scenarios where explicit judgment of edge existence is required. The combined use of the two metrics can comprehensively avoid the one-sidedness of a single metric; therefore, AUC and F1-Score were selected as the evaluation metrics in this paper. Both AUC and F1-Score were computed on the combined test set, i.e., micro-aggregation across all edge types.

### 4.4. Experiment Result Analysis

This section compares the AUC and F1-Score of the proposed VE-HGCN model with those of the baseline models on the DBLP1, DBLP2, IMDB, and LAST.FM datasets. The experimental results are presented in Table 3, where the bolded values represent the optimal results for each evaluation metric.

The experimental results demonstrate that the proposed method outperforms all baseline algorithms in both AUC and F1-Score, exhibiting significant advantages. Specifically, on the DBLP1 dataset, the AUC and F1-Score of the VE-HGCN method improve by 3.59% to 52.07% and 7.78% to 34.37%, respectively; on the DBLP2 dataset, the corresponding improvements of the VE-HGCN in AUC and F1-Score range from 3.45% to 45.22% and from 6.96% to 42.48%, respectively; on the IMDB dataset, the AUC and F1-Score of the VE-HGCN increase by 0.18% to 43.56% and 0.56% to 18.96%, respectively; on the LAST.FM dataset, the VE-HGCN method achieves improvements of 2.14% to 33.57% in AUC and 2.76% to 25.38% in F1-Score.

As an unsupervised low-order network embedding method based on random walks, LINE learns node embeddings solely through low-order similarity. It neither addresses the type differences in heterogeneous networks nor captures high-order structures, thus yielding inferior experimental results compared with the proposed model across all datasets. Metapath2vec typically learns node embeddings based on a single meta-path; its random walk only captures sequential low-order correlations along the predefined meta-path and fails to exploit the high-order structural features of heterogeneous networks, which highlights the limitations of Metapath2vec in complex heterogeneous networks. The Meta-GNN is also a meta-path-guided heterogeneous graph learning method, but it improves network coverage by increasing the number of meta-path types and takes high-order information into account. Its performance is highly dependent on meta-path design. On the IMDB dataset, the predefined meta-paths match the semantic characteristics of the dataset well, resulting in an AUC close to that of the GCN, which demonstrates the capability of meta-paths to capture heterogeneous semantics. However, meta-path construction relies on domain prior knowledge, and the complex semantics of DBLP and LAST.FM cannot be fully covered by a limited number of meta-paths, leading to mediocre performance of the Meta-GNN on these two datasets. As a classic homogeneous graph convolution model, the GCN excels at aggregating local neighborhood information in homogeneous or low-heterogeneity networks. It is worth noting that although the IMDB dataset has more node and edge types than DBLP1 and DBLP2, connections almost only occur between nodes of the same type. The network structure is thus closer to homogeneity, with minimal cross-type interactions, essentially behaving as a collection of isolated homogeneous subgraphs. The GCN’s first-order neighborhood convolution can efficiently capture node feature dependencies, which explains why the performance of the VE-HGCN is very close to that of the GCN on this dataset. In contrast, for highly heterogeneous networks such as DBLP and LAST.FM, the GCN cannot distinguish the semantic differences between nodes and edges of different types, and its aggregation process introduces heterogeneous noise, resulting in performance inferior to that of the VE-HGCN. Compared with the Meta-GNN, the HAN is more flexible: it implements adaptive weighting of heterogeneous semantics by introducing node-level and semantic-level attention mechanisms. The overall experimental results of the HAN outperform those of the Meta-GNN, which reflects the advantages of the attention mechanism. Nevertheless, the HAN still relies on binary graph structures and cannot model high-order correlations; hence, its performance is inferior to that of the VE-HGCN. Moreover, the attention mechanism is susceptible to noisy data, making the HAN less stable than the VE-HGCN. HGCL optimizes node embeddings by maximizing the consistency between augmented graph views. But, HGCL is originally tailored for recommendation scenarios and focuses on preserving pairwise structural similarity instead of mining high-order heterogeneous hypergraph patterns; it also lacks a multi-view fusion mechanism to integrate diverse semantic information. Additionally, HGCL shows a larger standard deviation on DBLP1 indicating weaker experimental stability. The ie-HGCN is an interaction-aware enhanced heterogeneous graph convolutional network that captures implicit node-edge interaction correlations to refine feature aggregation. As the most competitive baseline, it achieves the second-best performance on DBLP1, DBLP2 and LAST.FM among all comparison methods. Nevertheless, the ie-HGCN relies on conventional heterogeneous graph extraction paradigms that restrict its capability in modeling complex high-order heterogeneous relationships, leading to consistent performance gaps against the VE-HGCN.

The method proposed in this paper uses hyperedges to preserve high-order relationships in heterogeneous information networks and employs a voting ensemble strategy to distinguish complex semantic information. As a result, it achieves favorable prediction results on all four datasets.

### 4.5. Analysis of Ablation Experiment Results 

To verify the impact of the adaptive soft-voting ensemble strategy on model performance, effectiveness experiments were conducted in this study on the ensemble module, with the design of ablation variants presented in Table 4.

Specifically, V1 is intended to validate the necessity of the ensemble strategy, V2 aims to demonstrate the superiority of soft voting over hard voting, V3 is designed to verify the performance gain brought by the adaptive weighting mechanism of soft voting, V4 is constructed to prove the necessity of FSP-based heterogeneous hypergraph construction by comparing it with traditional meta-path-based heterogeneous hypergraph construction, and V5 is developed to verify the advantage of the HGCN in capturing high-order structural information compared with classic heterogeneous Graph Convolutional Network (GCN).

**Figure 4:**
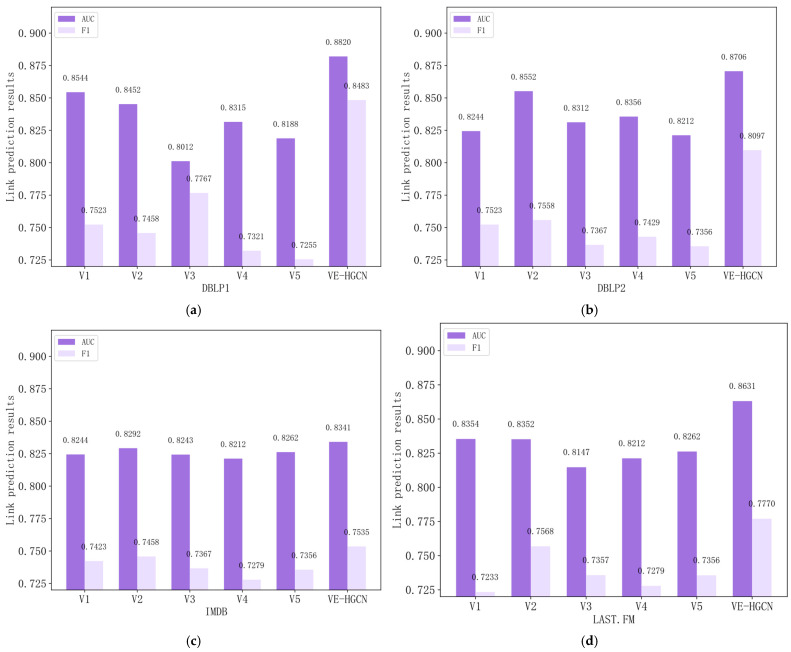
This figure illustrates the link prediction performance (AUC and F1-Score) of ablation variants, compared with the complete VE-HGCN model, across four different heterogeneous networks. (**a**) The link prediction performance of ablation variants in the DBLP1 network. (**b**) The link prediction performance of ablation variants in the DBLP2 network. (**c**) The link prediction performance of ablation variants in the IMDB network. (**d**) The link prediction performance of ablation variants in the LAST.FM network.

Based on the ablation experiment results presented in Figure 4, we can draw the following conclusions.

1. V1 removes the ensemble strategy and uses only the single optimal base model for prediction. The model’s AUC and F1-Score show significant declines across all datasets. This is because a single base model can only capture the high-order semantic information contained in one type of hypergraph and cannot integrate the differentiated features of multiple hypergraphs, thus losing adaptability to complex heterogeneous networks. This fully verifies the necessity of the ensemble strategy for improving model performance.
2. V2 replaces soft voting with hard voting, and its performance is inferior to that of the complete VE-HGCN. This is because hard voting only uses the category proportion of branch prediction results as the decision basis, ignoring the confidence differences in the prediction probabilities of different hypergraph branches, and thus cannot accurately distinguish the semantic contribution of each branch. This demonstrates the superiority of soft voting over hard voting.
3. V3 replaces adaptive weighted soft voting with equal-weighted averaging, and the model performance also declines. This is because equal-weighted averaging assumes that all hypergraph branches are equally important and cannot dynamically adjust weights according to the heterogeneity characteristics of different datasets. In contrast, the adaptive weighting mechanism can specifically strengthen the role of high-contribution hypergraphs and weaken the interference of low-efficiency branches, verifying the performance gain of the adaptive weighting mechanism.
4. V4 replaces FSP-based heterogeneous hypergraph construction with traditional meta-path-based construction. Its AUC and F1-Score drop obviously across all datasets, and V4 performs consistently worse than the full VE-HGCN despite minor fluctuations on individual datasets. Traditional meta-paths are manually predefined linear patterns that only capture limited pairwise node relations, failing to mine implicit non-linear high-order heterogeneous structures. In contrast, FSP-based construction is data-driven and automatically extracts fine-grained high-order patterns without manual priors, fully demonstrating its necessity and superiority in modeling heterogeneous network structures.
5. V5 replaces the HGCN with the classic heterogeneous GCN while keeping other modules unchanged. It underperforms V4 in most cases and shows a significant performance gap against the VE-HGCN on all four datasets. The classic heterogeneous GCN is limited to pairwise node relation modeling and cannot effectively encode the multi-node high-order correlations captured by FSP hypergraphs. This verifies the HGCN’s distinct advantages in processing high-order heterogeneous information and its indispensable role in the VE-HGCN.
6. The complete ablation experiment evaluation results verify the effectiveness of each component in the ensemble module. The adaptive weighted soft-voting mechanism is the core of the ensemble strategy and has the greatest impact on the model’s link prediction performance. Meanwhile, the introduction of the ensemble strategy and the replacement of hard voting with soft voting also play key roles in enhancing the robustness and accuracy of the model. In addition, the FSP-based heterogeneous hypergraph construction and HGCN-based high-order feature learning are two fundamental pillars of the model. The former provides high-quality high-order structural features, and the latter efficiently encodes these features. The synergistic effect of all components enables the complete VE-HGCN to achieve the optimal performance on all datasets, fully proving the rationality and advancement of the model design.

### 4.6. Validity Study of FSPs

**Figure 5:**
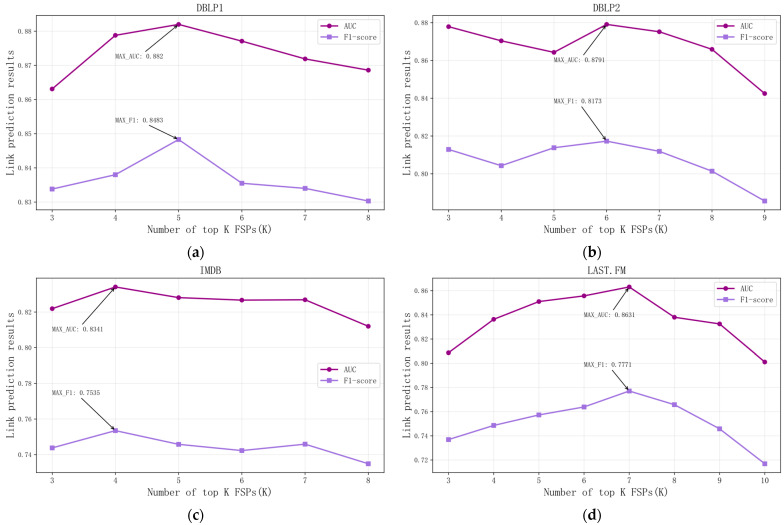
This figure illustrates the link prediction performance of the VE-HGCN model with different numbers of extracted FSPs across four heterogeneous networks: (**a**) shows the link prediction performance on the DBLP1 dataset; (**b**) on the DBLP2 dataset; (**c**) on the IMDB dataset; and (**d**) on the LAST.FM dataset.

To thoroughly investigate the impact of the number and type of FSPs extracted during the hypergraph construction phase on the link prediction performance of the VE-HGCN model, experiments were conducted on four datasets, namely, DBLP1, DBLP2, IMDB, and LAST.FM. The number of extracted FSPs was set in a differentiated manner according to the structural characteristics of different datasets. The link prediction performance of the model under different FSP numbers was systematically compared using the AUC metric and F1-Score, with the experimental results shown in Figure 5.

From the experimental results in Figure 5, it can be observed that the model’s performance is not proportional to the number of hypergraphs constructed—more hypergraphs do not equate to better performance; across different datasets, the optimal number and type of FSPs vary on the DBLP1 dataset, and the performance is optimal when 5 FSPs are extracted. On the DBLP2 dataset, the optimal number of extracted FSPs is 6, and on the IMDB dataset, the optimal number of extracted FSPs is 4, and the performance fluctuation is small as the FSP count changes. This is related to the fact that the network structure of the IMDB dataset is closer to a homogeneous network, resulting in fewer differentiable FSP features that can be extracted, and on the LAST.FM dataset, the performance is optimal when 7 FSPs are extracted compared with other datasets, LAST.FM requires more FSPs to cover complex heterogeneous semantic relationships; in summary, hypergraph construction needs to match the structural characteristics of the dataset, rather than simply increasing the number of FSPs.

**Figure 6:**
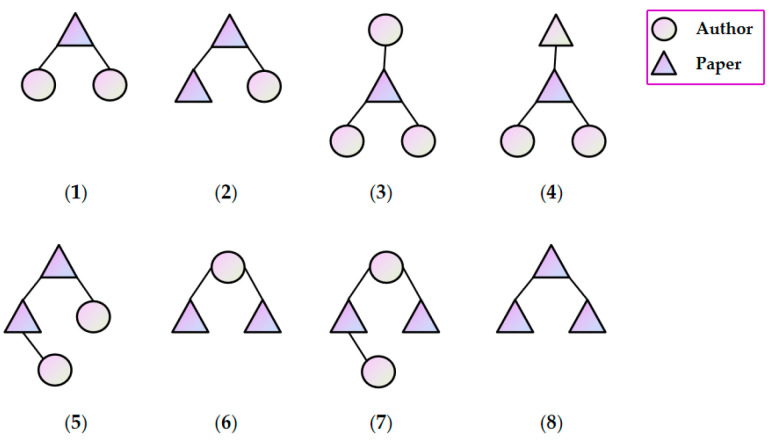
This figure illustrates the detailed structures of the top 8 most frequently occurring FSPs extracted from the DBLP1 dataset composed of author and paper nodes.

Taking the DBLP1 dataset as an example, the FSPs extracted from the DBLP1 dataset are visualized in Figure 6. The DBLP1 dataset contains only two types of nodes (papers and authors) and three typical relationships (author–paper, paper–paper, and author–author). As shown in Figure 6, (1)–(8) represent the top 8 most frequently occurring FSPs extracted from the DBLP1 dataset, with occurrence frequencies of 15,688, 13,774, 12,883, 10,870, 6982, 3414, 3030 and 2545 respectively. The experimental results of the DBLP1 dataset in Figure 6 indicate that the link prediction performance is optimal when hypergraphs are constructed using FSPs (1)–(5). This aligns well with the high-order structural characteristics of DBLP1. When more FSP structures are introduced, the performance begins to decline. The reason for this is that incorporating more FSP structures or mapping longer dependency paths into hyperedges tends to include weakly correlated relationships, introducing noise and redundancy. Meanwhile, larger-scale FSPs are sparser in real networks, leading to over-smoothing of information transmission during diffusion; additionally, the increase in parallel paths raises the model capacity and training difficulty, which can compromise generalization when the sample size is limited. Therefore, in the DBLP1 network dataset, which has only two node types but rich triangular and closed-loop structures, constructing hypergraphs with (1)–(5) and combining them with soft-voting fusion is sufficient to capture comprehensive and effective high-order structures, representing the most robust trade-off between efficiency and performance. For graphs with sparse high-order structures and dominated by pairwise relationships, such as the IMDB dataset, the FSP scale should be reduced, or hybrid modeling with binary graph convolution should be adopted to avoid redundancy and instability introduced by hypergraphs.

### 4.7. Parameter Sensitivity Analysis

To thoroughly investigate the impact of key hyperparameters on model performance, we conducted comprehensive sensitivity analysis on the number of HGCN layers, number of sampled subgraphs and subgraph expansion probability.

**Figure 7:**
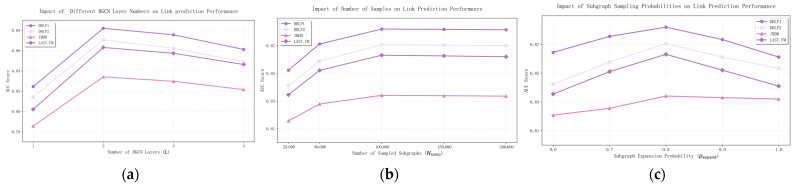
This figure illustrates impact of key hyperparameters on link prediction performance (AUC): (**a**) shows the impact of different HGCN layer numbers on link prediction performance; (**b**) the impact of subgraph sample numbers; and (**c**) the impact of subgraph expansion probability.

According to the experiment results represented in Figure 7, we can see 2 layers achieve optimal performance across most datasets. Additional layers lead to over-smoothing of node representations, where nodes become indistinguishable. Single-layer models cannot capture sufficient high-order neighborhood information. Performance improves with more samples up to 100,000 then plateaus. This indicates that 100,000 samples provide sufficient coverage of the pattern space for these datasets. Analysis: The 0.8/0.2 split between expansion and jump probabilities achieves optimal balance. Higher expansion probability ensures connected subgraphs, while some jump probability maintains sampling diversity and prevents bias toward high-degree regions.

### 4.8. Time Complexity Analysis and Training Time Analysis

To investigate the prediction efficiency of the VE-HGCN, time complexity analysis and comparative experiments on training time with baseline methods were performed in this study. Table 5 and Table 6 show the results of the time complexity analysis and training time comparison.

## 5. Conclusions

To address the problems that existing methods for heterogeneous network link prediction struggle to simultaneously capture high-order semantic structures and network heterogeneity, along with the limited robustness of single-model prediction, this paper proposes a VE-HGCN model for heterogeneous information network link prediction. Through the synergistic operation of three modules—heterogeneous hypergraph construction, feature learning, and voting ensemble—the model achieves dual improvements in both link prediction accuracy and stability. The heterogeneous hypergraph construction module extracts high-frequency FSPs and maps them to hyperedges, which not only preserves the high-order correlation features of the heterogeneous network but also maintains network heterogeneity. The feature learning module takes the HGCN as the core, excavates complex structural information in multiplex hypergraphs, and outputs multiple sets of preliminary prediction probabilities. The voting ensemble module integrates these multiple sets of results via a soft-voting strategy, distinguishes the diverse high-order information contained in multiplex hypergraphs, and mitigates the bias of single-branch predictions. Experimental analyses on four real-world network datasets demonstrate that the VE-HGCN model can effectively extract and utilize high-order information for link prediction, exhibiting great potential especially in the case of highly heterogeneous networks. These results verify that the link prediction performance of the method proposed in this paper outperforms that of the compared baseline methods.

Although the VE-HGCN model demonstrates excellent performance in improving link prediction accuracy, we must acknowledge that the computational complexity of hypergraph construction and convolution increases linearly with network size, resulting in an efficiency bottleneck in ultra-large-scale yet sparse heterogeneous networks. If we can strike an optimal balance between improving method accuracy and reducing its time complexity, the capability and applicability of the VE-HGCN will be further enhanced. Therefore, exploring more efficient feature extraction techniques and developing models that can simultaneously leverage low-order and high-order information will be the key directions for our future research.

## Figures and Tables

**Table 1:** Topological features of datasets. N represents the total number of nodes in the network, and E represents the total number of edges. N_type indicates the number of node types, and E_type stands for the number of edge types.

Network	N	E	N_Type	E_Type
DBLP1	6619	11,976	2	3
DBLP2	13,891	342,56	3	5
IMDB	31,669	147,128	5	5
LAST.FM	128,705	375,114	4	5

**Table 2:** Complete hyperparameter configuration.

Hyperparameter	Value	Description
${num}_{samples}$	100,000	Number of random subgraph samples for pattern extraction
${size}_{FSP}$	3, 4, 5	Target size of subgraph patterns
$p_{expand}$	0.8	Probability of neighborhood expansion
$p_{jump}$	0.2	Probability of global random jump
η	0.01	Adam optimizer learning rate
L	2	The number of hypergraph convolutional layers
$d_{1}$	64	First HGCN layer dimension
$d_{2}$	32	Second HGCN layer dimension
$d_{fc}$	64	Fully connected layer dimension
$p_{drop}$	0.2	Dropout rate for regularization
λ	0.0001	L2 regularization coefficient
$E_{pat}$	50	Epochs without improvement for early stopping
$\delta_{F1}$	0.001	Minimum F1-Score improvement threshold
$r_{neg/pos}$	1:1	Ratio of negative to positive samples

**Table 3:** Comparison of link prediction performance of various models on different datasets. The bolded parts in the table footer represent the proposed model and its corresponding performance.

Metrics	AUC				F1-Score			
Model Datasets	DBLP1	DBLP2	IMDB	LAST.FM	DBLP1	DBLP2	IMDB	LAST.FM
LINE	0.7281 ± 0.0015	0.6583 ± 0.0029	0.5810 ± 0.0017	0.6462 ± 0.0144	0.6821 ± 0.0028	0.6524 ± 0.0022	0.6334 ± 0.0003	0.6197 ± 0.0003
Metapath2vec	0.5800 ± 0.0045	0.5995 ± 0.0038	0.7441 ± 0.0032	0.7820 ± 0.0041	0.6313 ± 0.0035	0.5683 ± 0.0028	0.7269 ± 0.0025	0.7293 ± 0.0030
Meta-GNN	0.6609 ± 0.0032	0.6536 ± 0.0097	0.8062 ± 0.0019	0.7343 ± 0.0023	0.6666 ± 0.0027	0.6070 ± 0.0062	0.7346 ± 0.0021	0.6829 ± 0.0020
GCN	0.8046 ± 0.0039	0.7894 ± 0.0041	0.8326 ± 0.0027	0.7746 ± 0.0030	0.7042 ± 0.0038	0.6804 ± 0.0021	0.7493 ± 0.0021	0.6751 ± 0.0025
HAN	0.6966 ± 0.0058	0.6867 ± 0.0033	0.8083 ± 0.0024	0.7436 ± 0.0059	0.6671 ± 0.0035	0.6709 ± 0.0020	0.7473 ± 0.0022	0.6831 ± 0.0050
HGCL	0.8395 ± 0.0100	0.8002 ± 0.0050	0.8134 ± 0.0027	0.8290 ± 0.0027	0.7797 ± 0.0019	0.7379 ± 0.0055	0.7260 ± 0.0027	0.7355 ± 0.0078
ie-HGCN	0.8514 ± 0.0064	0.8416 ± 0.0084	0.8110 ± 0.0035	0.8450 ± 0.0081	0.7871 ± 0.0088	0.7570 ± 0.0040	0.7378 ± 0.0053	0.7561 ± 0.0086
**VE-HGCN**	**0.8820** ± 0.0035	**0.8706** ± 0.0042	**0.8341** ± 0.0028	**0.8631** ± 0.0031	**0.8483** ± 0.0040	**0.8097** ± 0.0038	**0.7535** ± 0.0025	**0.7770** ± 0.0033

**Table 4:** Ablation variants.

Variant ID	Operation
V1	Remove the ensemble strategy, use only the single optimal base model.
V2	Replace soft voting with hard voting, majority rule.
V3	Replace soft voting with equal-weighted averaging.
V4	Replace FSP hypergraph construction with meta-path-based hypergraph construction
V5	Replace the HGCN with the classic heterogeneous GCN

**Table 5:** Theoretical time complexity. K is the number of patterns, |Ehyper| is the average number of hyperedges per hypergraph, d is the hidden dimension, and epochs is the number of training iterations.

Component	Time Complexity
Hypergraph Construction	O(*K × |E_train_|*)
HGCN Forward Pass (Per Epoch)	O(*K* × (|*V*| + |*E_hyper_*|) × *d*).
Ensemble Weight Learning	O(*K × |E_val_|*)
Total Training	O(${num}_{samples}\times {size}_{FSP}^{2}$*+ K × |E| × epochs × d*)

**Table 6:** Training time comparison (seconds). The results are reported as mean ± standard deviation over 5 independent runs. And the training time includes data preprocessing, model training, and validation. The bolded parts in the table footer represent the proposed model and its training time for different datasets.

Model Datasets	DBLP1	DBLP2	IMDB	LAST.FM
LINE	4.45 ± 0.09	6.64 ± 0.11	6.13 ± 0.10	2.04 ± 0.06
Metapath2vec	5.22 ± 0.12	7.86 ± 0.15	7.31 ± 0.13	2.87 ± 0.09
Meta-GNN	1.31 ± 0.03	1.01 ± 0.02	1.04 ± 0.02	1.00 ± 0.01
GCN	1.80 ± 0.04	0.95 ± 0.02	0.95 ± 0.02	0.90 ± 0.01
HAN	1.13 ± 0.03	1.38 ± 0.04	1.30 ± 0.03	1.76 ± 0.05
HGCL	4.19 ± 0.22	14.45 ± 0.38	10.71 ± 0.32	11.65 ± 0.35
ie-HGCN	4.25 ± 0.18	9.34 ± 0.26	9.24 ± 0.25	7.85 ± 0.21
**VE-HGCN**	**3.16 ± 0.15**	**8.42 ± 0.29**	**7.34 ± 0.27**	**6.09 ± 0.26**

## Data Availability

The data presented in this study are available on request from the corresponding author.
